# Transcatheter aortic valve replacement with JenaValve Trilogy system for aortic regurgitation following a David procedure: a case report

**DOI:** 10.1093/ehjcr/ytaf450

**Published:** 2025-09-16

**Authors:** Jeng-Wei Chen, Tsung-Yo Ko, Ying-Hsien Chen, Chih-Yang Chan, Mao-Shin Lin

**Affiliations:** Department of Surgery, National Taiwan University Hospital, Taipei 100225, Taiwan; Department of Internal Medicine, National Taiwan University Hospital, Taipei 100225, Taiwan; Department of Internal Medicine, National Taiwan University Hospital, Taipei 100225, Taiwan; Department of Surgery, National Taiwan University Hospital, Taipei 100225, Taiwan; Department of Internal Medicine, National Taiwan University Hospital, Taipei 100225, Taiwan

**Keywords:** Aortic regurgitation, Case report, Transcatheter aortic valve replacement, Valve-sparing aortic root replacement

## Abstract

**Background:**

Valve-sparing aortic root replacement (VSARR), also known as the David procedure, is a preferred surgical option for patients with aortic root aneurysms, offering preservation of the native valve and avoidance of mechanical valve replacement with lifelong anticoagulation. However, long-term durability remains a concern, with progressive aortic regurgitation (AR) occurring in up to 20% of cases. Redo surgical valve replacement is standard for failed VSARR, but transcatheter aortic valve replacement (TAVR) has emerged as a less invasive alternative for high-risk patients, despite technical challenges in non-calcified anatomy. This case highlights the first reported use of the JenaValve Trilogy system to treat post-David procedure AR.

**Case summary:**

A 71-year-old female developed severe AR ten years after undergoing a David procedure for a sinus of Valsalva aneurysm. Echocardiography showed eccentric AR due to left and non-coronary cusp malcoaptation, with an effective regurgitant orifice of 0.28 cm². Due to comorbidities and patient preference, TAVR was selected over surgical redo. A 23-mm Trilogy JenaValve was implanted via transfemoral access under conscious sedation. Post-procedural aortogram confirmed successful deployment and absence of residual regurgitation. The patient recovered uneventfully and was discharged on postoperative day five.

**Discussion:**

This case demonstrates the feasibility of the JenaValve Trilogy for managing post-VSARR AR, even in the absence of annular calcification. Its unique anchoring mechanism, which uses locators positioned within the sinuses, provides secure fixation in complex anatomies. This leaflet-locating design may offer a transcatheter option for patients with non-calcified anatomy. While promising, further clinical experience and long-term data are needed to better define the role of this system in failed valve-sparing repairs.

Learning points

- The JenaValve Triology can be safely used for AR after failed VSARR, might offering a transcatheter option in non-calcified anatomy where traditional TAVR devices are challenging.
- Successful valve deployment requires precise alignment, especially in cases with leaflet shrinkage and eccentric regurgitation after the David procedure.

## Introduction

The David procedure, a valve-sparing aortic root replacement (VSARR), is commonly performed for aortic root aneurysms. However, progressive aortic regurgitation (AR) can develop over time, necessitating reintervention. Transcatheter aortic valve replacement (TAVR) has emerged as a viable alternative for high-risk surgical candidates. Treatment of pure AR with conventional TAVR prostheses is challenging due to the lack of annular calcification, which can lead to a higher risk of device malposition or embolization. The JenaValve Trilogy, with its unique anchoring mechanism, is CE-marked for native pure AR and demonstrates superior acute performance compared to other transcatheter heart valves.^1^ While previous reports have demonstrated the use of SAPIEN or Evolut valve system in this scenario, this case describes the first successful implantation of a JenaValve Trilogy to treat post-David procedure AR.

## Summary figure

**Figure:**
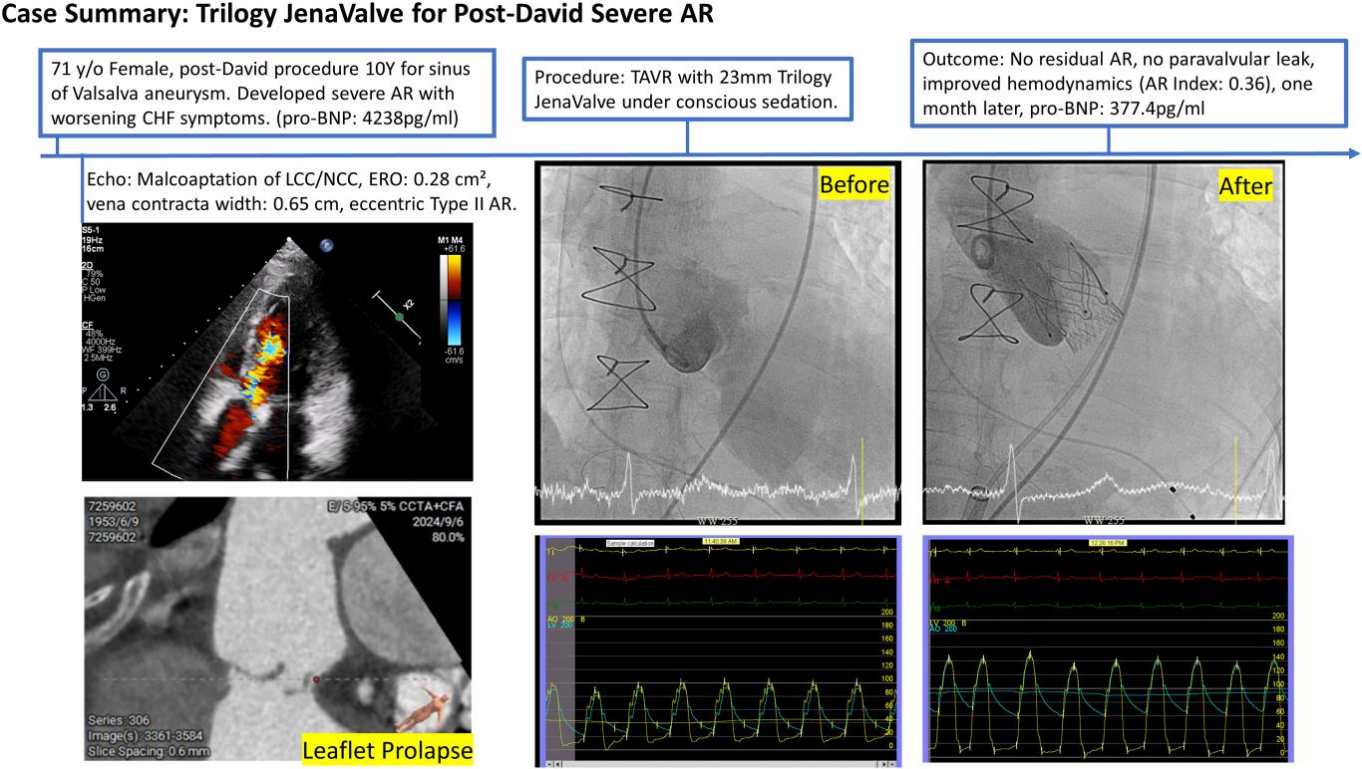


## Case Presentation

**Figure 1:**
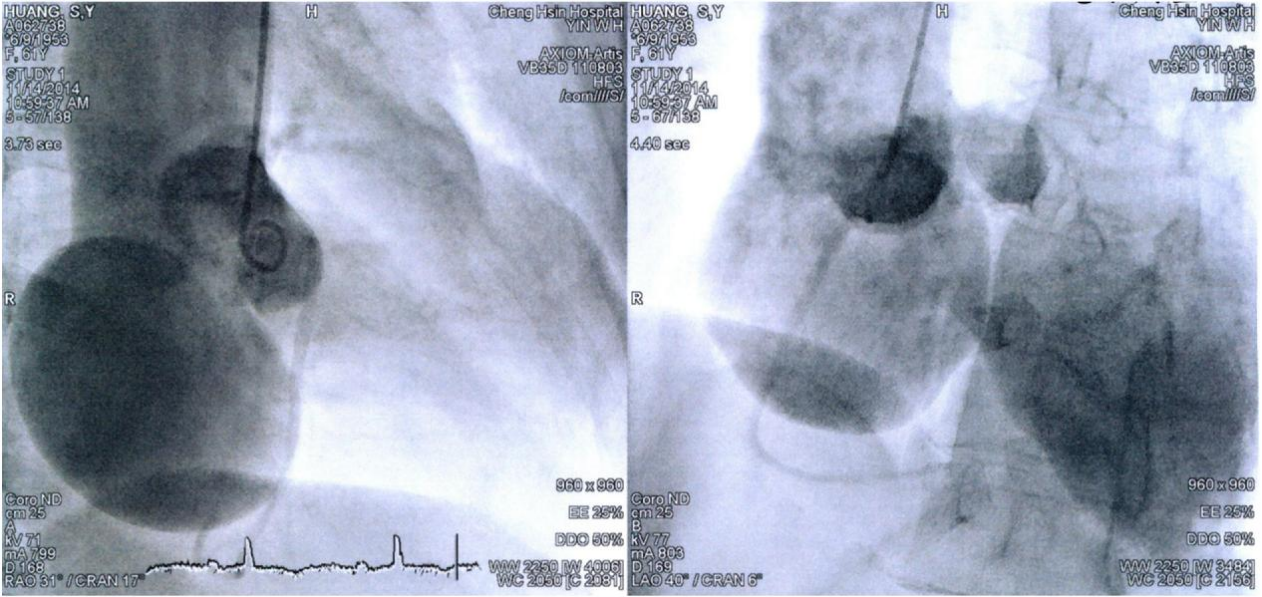
Sinus of Valsalva aneurysm before valve-sparing aortic root replacement (VSARR). The native aortic valve was trileaflet, with a large non-coronary cusp (NCC) aneurysm measuring over 5 cm.

**Figure 2:**
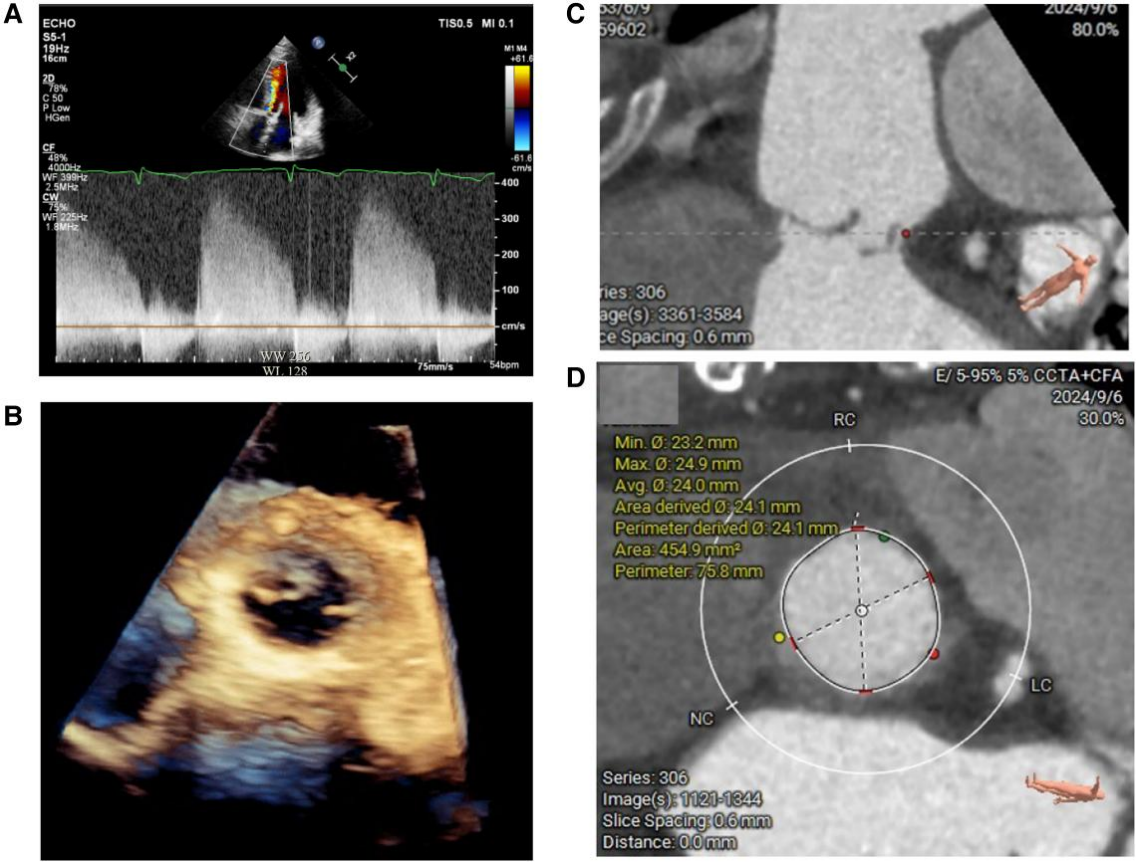
(*A*) Transthoracic echocardiography demonstrating severe eccentric aortic regurgitation in the apical four-chamber view. (*B*) Three-dimensional transoesophageal echocardiography (3D-TEE) showing shrinkage of the non-coronary cusp and malcoaptation between the non-coronary and left coronary cusps. (*C*) Preoperative computed tomography (CT) image illustrating prolapse of the non-coronary cusp with incomplete coaptation. (*D*) Preoperative CT annular assessment at 30% systolic phase, showing an annular perimeter of 75.8 mm.

A 71-year-old woman with a history of sinus of Valsalva aneurysm, involving dilation confined to the non-coronary cusp side and a trileaflet aortic valve (*Figure 1*), underwent a David procedure 10 years earlier, including reconstruction with a 28-mm Valsalva Dacron graft. Serial echocardiography revealed progressive aortic regurgitation (AR), and over the past year, she developed worsening exertional dyspnoea. Her pro-B-type natriuretic peptide (pro-BNP) level was markedly elevated at 4238 pg/mL. Initial medical management with diuretics led to pre-renal azotaemia. Follow-up echocardiography demonstrated left coronary artery cuspid prolapse with malcoaptation of the left and non-coronary cusps, resulting in eccentric moderate-to-severe type II AR. Quantitative assessment revealed an effective regurgitant orifice area of 0.28 cm², a regurgitant volume of 53.1 mL, and a vena contracta width of 0.65 cm. Additional findings included left ventricular dilation (LVEDD 40 *mm/m*² *BSA*; LVESD 26 *mm/m*² *BSA*) and holodiastolic flow reversal, consistent with significant haemodynamic burden (*Figure 2*). Given the severity of AR, reintervention was recommended. Although the EuroSCORE II was 7.2% and the cardiac surgeon recommended surgical AVR based on her age, the patient declined redo surgery. Following a Heart Team discussion, TAVR was selected as the treatment approach.

**Figure 3:**
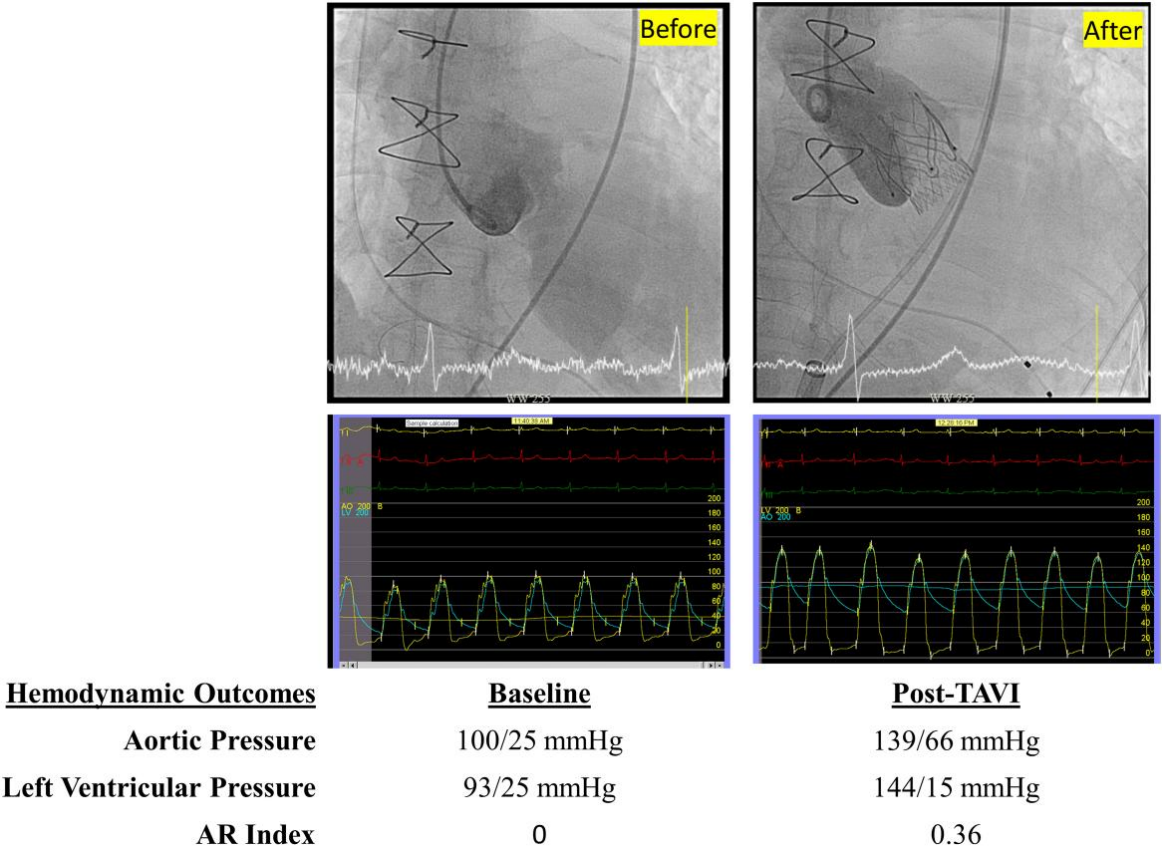
Fluoroscopic images and haemodynamic tracings before and after TAVR with the Trilogy JenaValve system. (Top) Angiography shows severe aortic regurgitation before TAVR (left) and no residual regurgitation after valve implantation (right). (Bottom) Pressure tracings demonstrate haemodynamic improvement. Aortic pressure improved from 100/25 mmHg to 139/66 mmHg, left ventricular pressure changed from 93/25 mmHg to 144/15 mmHg, and the AR index increased from 0 to 0.36 after TAVR.

Preprocedural imaging during the 30% systolic phase showed an annular perimeter of 75.8 mm and a left ventricular outflow tract (LVOT) measurement of 77.5 mm. A 23-mm Trilogy JenaValve was selected (oversizing, 5.0–13.5%). Under conscious sedation, the TAVR was delivered from the right femoral artery via a 20 Fr sheath following a preset ProGlide puncture-site closure system. A temporary pacemaker was inserted via the right internal jugular vein, and a 6Fr sheath was set up at the left femoral for a pigtail catheter. The valve was deployed successfully without complications. Post-procedural angiography confirmed no residual AR (*Figure 3*, Supplementary material online, *Video*). Diastolic pressure improved from 25 to 66 mmHg. The temporary pacemaker was removed immediately after the procedure, as sinus rhythm was maintained throughout.

The patient recovered uneventfully and was discharged on postoperative day five. At the one-month follow-up, her symptoms had completely resolved. The patient remained in sinus rhythm. The pro-BNP level had decreased to 377.4 pg/mL and remained stable at 489.1 pg/mL at three months. Echocardiography at 3 months showed no evidence of paravalvular leak or residual aortic regurgitation. The transaortic valve mean pressure gradient was 7.2-mmHg. LVEF was preserved at 61%, and left ventricular dimensions demonstrated favourable reverse remodelling, with LVEDD 30 mm/m² BSA and LVESD 21 mm/m² BSA.

## Discussion

VSARR has become a standard procedure for young patients with aortic regurgitation, offering the advantage of preserving the native valve and avoiding lifelong anticoagulation required with mechanical valve replacement. By eliminating the risks associated with mechanical prostheses, such as thromboembolic events and endocarditis, VSARR is a preferred option for select patients. However, despite its benefits, long-term durability remains a concern. Even in experienced hands, the incidence of severe aortic insufficiency following VSARR has been reported at approximately 10% at 5 years and 15% at 10 years, with up to 20% of patients requiring reintervention due to valve failure.^2^

While redoing surgical aortic valve replacement (SAVR) via resternotomy remains the gold standard with relatively low surgical risk, some patients with severe comorbidities may not be suitable candidates for another open-heart procedure.^3^ In such cases, TAVR has emerged as a potential alternative, although its use in failed VSARR is currently off-label. Previous reports have primarily demonstrated the successful implantation of SAPIEN 3 or Evolut R CoreValve in cases of failed VSARR procedures. However, TAVR in this setting presents unique technical challenges due to the lack of annular calcification, making it difficult to anchor the transcatheter valve securely.^4^

Traditionally, the subvalvular pledgeted sutures used in David procedures have been proposed as a stable anchoring platform for TAVR valves. Theoretically, these sutures could provide a secure fixation point for either balloon-expandable or self-expanding valves.^5^ Additionally, over time, fibrosis or scarring along the suture plane may enhance friction, further stabilizing the transcatheter valve. Isao *et al*. previously reported a successful case utilizing the residual cord-knot radiopaque markers as anchoring landmarks for an SAPIEN 3 valve.^6^

The JenaValve Trilogy system has a unique mechanism, fixating with locators within the sinuses, making it a viable option for treating pure aortic regurgitation in the absence of valve calcification. The ALIGN-AR study demonstrated the safety and efficacy of the Trilogy valve, with promising 30-day and one-year outcomes in inoperable patients with severe AR.^7^ In our case, we present the first documented use of the JenaValve Trilogy system to treat a failed VSARR, demonstrating its feasibility and excellent haemodynamic performance.

However, challenges remain. Post-David procedure failures often involve leaflet shrinkage, leading to malcoaptation and severe regurgitation.^8^ In our case, the shrinkage of the non-coronary cusp with concomitant left coronary cusp prolapse and a relatively horizontal aortic root created an eccentric AR jet and complicated the positioning of the Trilogy valve. Achieving correct locator placement required three to four recapture and repositioning attempts, with particular attention to engaging the left and non-coronary cusps first due to the longest anchoring distance. We optimized fluoroscopic angulation and used intra-procedural transoesophageal echocardiography to confirm that all locators were seated within the respective sinuses. Once proper alignment was obtained, ensuring the sealing ring was flush with the anchor plane was essential for procedural success.

Lifetime management is a critical consideration in patients with failed aortic root procedures, particularly among younger and low-risk individuals. Patients with isolated aortic regurgitation often present with annular dilation, necessitating the use of larger valve sizes. The JenaValve system, with its short stent frame, potentially offers a lower risk of coronary obstruction in future valve-in-valve (TAVR-in-TAVR) interventions. While the short-term outcome in our case was favourable, the long-term durability of the transcatheter valve remains uncertain. Younger patients may still benefit from surgical reintervention due to their longer life expectancy and generally acceptable risk profile for redo surgery. This case illustrates how patient age, anatomical features, and procedural strategy must be carefully weighed in constructing a tailored lifetime treatment plan, in line with recent perspectives on long-term aortic valve.^9^

## Conclusion

This case illustrates the first successful TAVR using the JenaValve Trilogy for post-David aortic insufficiency, demonstrating its safety, feasibility, and effectiveness. Given the increasing need for transcatheter solutions in failed VSARR, the JenaValve system presents a promising alternative. Further research is warranted to establish its role in similar cases.

## Lead author biography


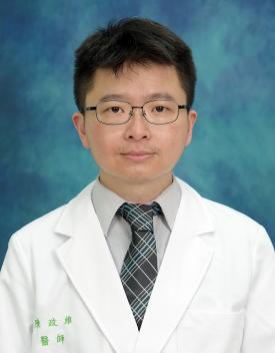


Dr Jeng-Wei Chen is an attending cardiovascular surgeon at National Taiwan University Hospital. His clinical and academic expertise spans adult cardiac surgery, mechanical circulatory support, transcatheter valve and aortic interventions, and cardiac gene therapy. Dr Chen completed his surgical residency and cardiovascular surgery training at NTUH. He later obtained a PhD in Clinical Medicine from National Taiwan University in 2022. He has also received international research training as a visiting fellow at the Duke University Medical Center=E2=80=99s Department of Cardiothoracic Surgery and Transplant Center (2023=E2=80=932024).

## Supplementary Material

ytaf450_Supplementary_Data

## Data Availability

The data underlying this article are available in the article and its online Supplementary material.
